# Influence of Cooking Methods on Onion Phenolic Compounds Bioaccessibility

**DOI:** 10.3390/foods10051023

**Published:** 2021-05-08

**Authors:** Alice Cattivelli, Angela Conte, Serena Martini, Davide Tagliazucchi

**Affiliations:** Department of Life Sciences, University of Modena and Reggio Emilia, 42100 Reggio Emilia, Italy; alice.cattivelli@unimore.it (A.C.); serena.martini@unimore.it (S.M.); davide.tagliazucchi@unimore.it (D.T.)

**Keywords:** mass spectrometry, food processing, metabolomics, polyphenols, in vitro digestion, thermal treatments

## Abstract

The impact of domestic cooking (baking, boiling, frying and grilling) and in vitro digestion on the stability and release of phenolic compounds from yellow-skinned (YSO) and red-skinned onions (RSO) have been evaluated. The mass spectrometry identification pointed out flavonols as the most representative phenolic class, led by quercetin-derivatives. RSO contained almost the double amount of phenolic compounds respect to YSO (50.12 and 27.42 mg/100 g, respectively). Baking, grilling and primarily frying resulted in an increased amount of total phenolic compounds, especially quercetin-derivatives, in both the onion varieties. Some treatments promoted the degradation of quercetin-3-*O*-hexoside-4′-*O*-hexoside, the main compound present in both the onion varieties, leading to the occurrence of quercetin-4′-*O*-hexoside and protocatechuic acid-4-*O*-hexoside. After in vitro digestion, the bioaccessibility index for total phenolic compounds ranged between 42.6% and 65.5% in grilled and baked YSO, respectively, and between 39.8% and 80.2% in boiled and baked RSO, respectively. Baking contributed to the highest amount of bioaccessible phenolic compounds for both the onion varieties after in vitro digestion. An in-depth design of the cooking process may be of paramount importance in modulating the gastro-intestinal release of onion phenolic compounds.

## 1. Introduction

Epidemiological data and human intervention trials have highlighted the protective effect of a polyphenol-rich food diet against the onset of chronic and non-communicable diseases including cardiovascular diseases and metabolic disorders [1,2]. Among fresh vegetables, onion was the second most cultivated, accounting for 8.1% of the European Union total fresh vegetables [3]. In recent years, several in vivo studies have described the beneficial effects of allium vegetables (including onion) consumption on cardiovascular diseases, hypertension and cancer [4,5].

The most important bioactive components found in onion are organosulfur and phenolic compounds, especially flavonols and, in the red varieties, anthocyanins [6,7].

Quercetin-derivatives are the major onion flavonols, with quercetin 3,4′-diglucoside and quercetin 4′-glucoside representing about the 90% of the total flavonols content [8]. Minor components such as kaempferol-and isorhamnetin-derivatives have also been detected in onion [6,7]. The onion flavonols content strongly depends on the variety. Red and yellow onion varieties may contain up to 1 g/kg of fresh weight of flavonols whereas the white onion variety displays a lower amount of flavonols (7 mg/kg) [6,8]. Red onion variety was also characterized by the presence of anthocyanins, mainly cyanidin-derivatives [7,9]. Flavonols have been reported to have a wide range of biological effects including antioxidant activity, protective effects against the onset of cardiovascular diseases, anti-diabetic and also anti-proliferative activities [6,10].

Onion can usually be consumed raw or following different cooking procedures such as boiling, frying, grilling and baking. Cooking processes may induce substantial changes on the physical structure of foods, which in turn result in a matrix softening effect due to a heat-induced breakdown of vegetables structure [11,12]. As a result, the extractability of phenolic compounds may increase after cooking due to the cell walls lysis and the release of fiber-bound phenolic compounds [11,12]. On the other hand, sometimes cooking may negatively affect the stability of phenolic compounds. Some phenolic structures may be thermolabile and are likely to experience thermal degradation during cooking [13]. Otherwise, hydrophilic phenolic compounds may be lost during cooking, especially during boiling, and released into cooking medium [11]. Cooking may also promote interactions between phenolic compounds and additional components in the food matrix such as proteins, fibers and lipids [14,15]. During high-temperature processes (such as grilling and baking) phenolic compounds may react with fibers and proteins in the Maillard reaction becoming part of the melanoidins structure and decreasing their content in the food [14,15].

The effect of cooking on phenolic compounds stability is dependent on a fine balance between their degradation or loss, their reaction with food matrix components and the increased extractability due to the matrix softening effect. This equilibrium strongly relies upon the cooking conditions (such as time, temperature and cooking medium), the food matrix and the phenolic structure [11,12].

Particular attention has been paid to the effect of cooking on onion phenolic compounds stability and extractability [16,17,18,19,20,21]. In all the studies, boiling was the thermal treatment that caused the highest loss of flavonols [17,18,19,20,21]. Conflicting results have been obtained when frying procedure was used. For example, Rodrigues et al. [17] did not find any effect of frying on flavonols content whereas Juaniz et al. [16] detected an increased extractability of flavonols after frying. By contrast, other authors found a small decrease in the onion flavonols content after frying [19,20]. Regarding the other cooking procedures, baking was found to have no or a slight negative effect on onion flavonols whereas grilling caused an increase in extractable flavonols concentration [16,17,21]. In red onion varieties, anthocyanins were always degraded independently of the thermal treatment but with different intensities [17].

To exert their positive health effects, phenolic compounds should be bioavailable, i.e., absorbed in the gastro-intestinal tract and reach the blood stream. The bioavailability of phenolic compounds strongly depends on their bioaccessibility, which can be defined as the fraction of phenolic compounds released from the food matrix in the gastro-intestinal tract [22]. Bioaccessibility quantification of phenolic compounds after digestion is of paramount importance for an appropriate evaluation of their intake, which is likely to be overestimated by considering only the mere phenolic content in the food [23]. Since the bioaccessibility is highly dependent on the phenolic compounds extractability, the different cooking procedures may have a pivotal impact on their bioaccessibility. For example, the application of distinct cooking methods resulted in a differential release of individual phenolic compounds in eggplant. Baked and grilled eggplant sample displayed the highest amount of bioaccessible caffeoylquinic acids whereas fried eggplant samples were rich in di-caffeoylquinic acids [13]. In this context, information on the effect of cooking on the bioaccessibility of onion phenolic compounds is still lacking.

Therefore, the objective of the present research was to assess the effect of the four different and most common cooking methods on the stability, bioaccessibility and antioxidant activity of phenolic compounds from two onion varieties (yellow-skinned and red-skinned onion) after cooking and in vitro gastro-intestinal digestion.

## 2. Materials and Methods

### 2.1. Materials

The reagents for total phenolic and antioxidant activity analysis as well as the materials and enzymes for the in vitro digestion were purchased from Sigma-Aldrich (Milan, Italy). Methanol, formic acid and acetonitrile for phenolic extraction and mass spectrometry analysis were obtained from BioRad (Hercules, CA, USA). Ferulic acid (catalogue number 52229), caffeic acid (catalogue number 51868), protocatechuic acid (catalogue number 03930590), quercetin-3-*O*-glucoside (catalogue number 16654), epicatechin (catalogue number 68097) and cyanidin-3-*O*-glucoside (catalogue number PHL89616) were supplied by Sigma-Aldrich (Milan, Italy). Yellow-skinned (YSO) and red-skinned onion (RSO) samples (*Allium cepa* L.) were purchased in a local supermarket (Reggio Emilia, Italy).

### 2.2. Onion Cooking

Four different procedures (i.e., baking, boiling, frying and grilling) were applied to cook onion. Each cooking experiment was carried out in three different trials as reported in Martini et al. [13]. Before cooking experiments, onions were peeled and cut longitudinally into thin slices of approximatively 0.3 cm. Briefly, for the baking procedure, onion was placed in a steel tray and cooked for 30 min at 180 °C in an electric oven. In the boiling treatment, onion was added to boiling tap water in a covered stainless-steel pot (1:5 onion/water ratio) and cooked for 30 min. After cooking, samples were drained for 30 s. Frying treatment was performed by adding onion to sunflower oil in a domestic deep fryer at 140 °C for 8 min. Grilling was carried out on a two-sided electric grill and the onion was cooked at 110 °C for 15 min. Before and after all the thermal treatments, the peeled and chopped onion was weighed to monitor the water loss caused by the cooking and samples stored at −80 °C until analysis.

All the cooking parameters are summarized in Table 1.

### 2.3. Preparation of Phenolic Compounds Methanol Extracts from Raw and Cooked YSO and RSO

The phenolic compounds extraction procedure was carried out following the method described by Martini, Conte, and Tagliazucchi [24]. Briefly, 15 g of raw and cooked onion samples were homogenized in 30 mL of a water/methanol/formic acid (28:70:2, *v*/*v*/*v*) solution. After 30 min of incubation at 37 °C, the homogenates were centrifuged (6000× *g*, 20 min, 4 °C) and the collected supernatant stored at −20 °C until further analysis.

### 2.4. In Vitro Gastro-Intestinal Digestion of Raw and Cooked Onion Samples

The in vitro digestion procedure was carried out applying the COST Action INFOGEST protocol [25]. The in vitro gastro-intestinal digestion comprised three sequential steps. The oral step was carried out by mixing 5 g of chopped raw or cooked onion with 5 mL of simulated salivary fluid added of 150 U/mL of salivary α-amylase. After homogenization, the oral bolus was incubated for 5 min at 37 °C in a rotating wheel (10 rpm). The gastric phase was performed by adding 10 mL of simulated gastric fluid (containing 2000 U/mL of pepsin) to the oral bolus and incubated for 2 h at 37 °C in a rotating wheel (10 rpm). Then, 15 mL of the intestinal fluid (containing pancreatin, 200 U/mL based on trypsin activity) were added to the gastric bolus, mimicking the final intestinal phase. After 2 h of incubation at 37 °C in a rotating wheel (10 rpm), an aliquot of the samples was withdrawn. After centrifugation (10,000× *g*; 20 min; 4 °C), the supernatants were collected and stored at −20 °C until further analysis.

The digestion experiments were performed in triplicate. 

Bioaccessibility index (BI) was determined as follow:

(1) $$\mathrm{Bioaccessibility}\;\left(\%\right)=\frac{\mathrm{Cd}}{\mathrm{Ce}}\times 100$$

Cd is the concentration of phenolic compounds in the supernatants collected at the end of the in vitro gastro-intestinal digestion whereas Ce is the concentration in the methanol/formic acid extract.

### 2.5. Determination of Total Phenolic Compounds and Antioxidant Activity in Raw and Cooked Onion Samples

Total phenolic compounds were quantified in the methanol extracts and in vitro digested samples of raw and cooked onion by using the Folin-Ciocalteu assay [26]. Data were reported as mg of gallic acid equivalent per 100 g of onion fresh weight. The antioxidant activity was measured as total radical scavenging activity and ferric reducing/antioxidant power by using the ABTS and FRAP assays, respectively [27,28]. Results were expressed as mg of ascorbic acid equivalent per 100 g of onion fresh weight.

### 2.6. Liquid Chromatography Electrospray Ionization Ion Trap Mass Spectrometry (LC-ESI-IT-MS) Profiling of Phenolic Compounds in Raw and Cooked Onion Samples

Phenolic compounds profiling of methanol/formic acid extracts and in vitro digested samples of raw and cooked onion was carried out by using a liquid chromatography system (HPLC Agilent 1200 Series, Agilent Technologies, Santa Clara, CA, USA) coupled with an Agilent 6300 ion trap mass spectrometer. Separation was performed with a C18 column (HxSil C18 Reversed phaseHamilton, 250 × 4.6 mm, 5 μm particle size, Hamilton Company, Reno, NV, USA) by using a binary gradient of water/formic acid and acetonitrile. The mobile phase composition, the flow rate, the elution gradient, the negative and positive ESI-MS parameters as well as the quantification method are fully described in Martini et al. [24,29] and Mekam et al. [30].

Ferulic acid-, caffeic acid- and protocatechuic acid-derivatives were quantified by using ferulic acid, caffeic acid and protocatechuic acid as standard references, respectively; flavonols and di-hydro-flavonols were quantified in quercetin-3-*O*-glucoside equivalents; flavan-3-ols were quantified by using epicatechin as standard compound; cyanidin-3-*O*-glucoside was used as reference compound for anthocyanins quantification.

### 2.7. Statistics

Each analysis was carried out in triplicates and data are shown as mean ± SD. One-way ANOVA with Tukey’s post-hoc test and Pearson correlation were performed by using Graph Pad prism 6.0 (GraphPad Software, San Diego, CA, U.S.A.) The differences were considered significant with *p* < 0.05.

## 3. Results and Discussion

### 3.1. Total Phenolic Content and Phenolic Profiles of YSO and RSO

A total of 13 and 28 phenolic compounds were identified and quantified in raw YSO and RSO samples, respectively. In particular, in YSO, 9 flavonols and 4 hydroxycinnamic acids were identified whereas in RSO, flavonols was the major representative class (14 individual compounds), followed by anthocyanins (8 individual compounds), di-hydro-flavonols (4 individual compounds) and flavan-3-ols (2 individual compounds). The compounds mass spectral data are reported in supplementary Tables S1 and S2, whereas the identified compounds, together with the quantitative data, are listed in Table 2 and Table 3. 

From a quantitative point of view, flavonols dominated the phenolic profiles of both the onion varieties, representing 94.1% and 74.2% of total phenolic compounds (Table 2 and Table 3).

Anthocyanins represented the 25.1% of total phenolic compounds in RSO whereas, in YSO, hydroxycinnamic acids accounted for the 5.9% of total phenolic compounds.

The most abundant compounds in both the onion varieties were quercetin-3-*O*-hexoside-4′-*O*-hexoside and quercetin-4′-*O*-hexoside, which together accounted for approximately 93.1% and 62.5% of total phenolic content in YSO and RSO, respectively.

These results complied with other authors’ studies [16,21,31] which reported percentages of quercetin mono- and di-hexoside between 80% and 93%, depending on the onion varieties.

Total phenolic content of YSO was roughly half the content of RSO, 27.42 ± 1.70 and 50.12 ± 0.65 mg/100 g of fresh weight, respectively (Table 2 and Table 3) as stated by previous studies [6,19]. The amount of onion phenolic compounds has been found to be strongly dependent on the onion variety, harvest year and meteorological conditions [17].

Similarly, the total flavonols content was 1.4 times higher in raw RSO compared to YSO (Table 2 and Table 3). Looking at the two most important compounds, the amount of quercetin-4′-*O*-hexoside was almost the double in RSO respect to YSO, whereas there were no differences in the amount of quercetin-3-*O*-hexoside-4′-*O*-hexoside between the two varieties (Table 2 and Table 3). 

The total amount of phenolic compounds measured with the Folin-Ciocalteu’s assay was 43.21 ± 1.57 and 65.44 ± 1.88 mg/100 g of fresh weight in YSO and RSO, respectively (Table 2 and Table 3). The total amount determined through mass spectrometry experiments was 64% and 77% of the Folin-Ciocalteu values in YSO and RSO, respectively.

### 3.2. Effect of Cooking Methods on Total and Individual Phenolic Content in YSO and RSO

Table 2 and Table 3 also show the effects of the different cooking treatments on the total and individual phenolic compounds in YSO and RSO. Considering the total amount of phenolic compounds (sum of the concentrations of the individual compounds identified by mass spectrometry), boiling was the only thermal treatment that caused a decrease in total phenolic content in both the onion varieties (37.5% and 13.9% decrease in YSO and RSO, respectively). On the contrary, frying was the cooking method that triggered the highest increase in total phenolic content determined by mass spectrometry (61.6% and 35.1% increase in YSO and RSO, respectively) followed by baking for YSO (58.7% increase) and grilling for the RSO (30.1% increase). These results were fairly different from those achieved with the Folin-Ciocalteu’s assay, where baking and grilling caused the highest increase in total phenolic content (Table 2 and Table 3). This discrepancy may be a consequence of the Maillard reaction, which occurs during cooking, generating compounds able to directly interact with the Folin-Ciocalteu’s reagent [32]. Supporting this, previous studies already reported that grilling and baking processes determined the formation of higher amounts of Maillard reaction products respect to frying and boiling in onion [33].

Similar results were obtained when considering the amount of total flavonols determined by mass spectrometry as a function of the cooking procedure (Table 2 and Table 3). The highest decrease in total flavonols was found for boiled (35.0% decrease) YSO whereas a marginal 1.4% decrease was observed in the boiled RSO. Once again, frying was the cooking method with the highest increase in total flavonols for both the varieties (68.5% and 57.1% increase in YSO and RSO, respectively).

In accordance, previous studies confirmed this trend of flavonols content dependent on different cooking processes. Specifically, flavonols concentration increased after grilling, baking and frying but decreased after boiling, compared to the raw samples [16,18,21,34]. Nevertheless, other studies found no effect or a small decrease in flavonols after frying. The variability in the reported data may be due to the different cooking parameters applied. It has been suggested that phenolic compounds stability and extractability after thermal treatments are strongly dependent on the cooking time and temperature as well as on the chopping procedure [11,35].

A significant increase of both quercetin-3-*O*-hexoside-4′-*O*-hexoside and quercetin-4′-*O*-hexoside was observed in the baked, fried and grilled YSO and RSO, compared to the raw samples (Table 2 and Table 3). In YSO, the percentage incidence of quercetin-di-hexosides on total phenolic compounds determined by mass spectrometry decreased from 75% in raw onion to 64%, 63% and 63% after baking, frying and grilling, respectively. In parallel, an increase in the quercetin-mono-hexosides percentage incidence was observed after baking, frying and grilling where they represented the 32%, 34% and 31% of total phenolic compounds, respectively, compared to the raw onion (percentage incidence of 18%) (Table 2). Vice versa, in boiled YSO, the percentage incidence of quercetin-di-hexosides increased, compared to the raw onion, reaching more than 90% of total phenolic compounds determined by mass spectrometry (Table 2). In RSO, the percentage incidence of quercetin-mono-hexosides was steady whatever the thermal treatment, whereas an increased incidence of quercetin-di-hexosides was observed after boiling and frying, compared to the raw onion (Table 3).

In YSO, the amount of total hydroxycinnamic acids determined by mass spectrometry decreased for all treatments with the exception of grilling where a 22.4% increase was obtained (Table 2). Boiling was responsible for the highest loss of total hydroxycinnamic acids (76.4% decrease). In RSO, grilling was the only treatment where the total amount of anthocyanins determined by mass spectrometry was similar to that of the raw sample (Table 3). In this case, baking caused the highest loss of total anthocyanins (84.7% decrease).

### 3.3. Effect of Cooking Methods on Total and Individual Phenolic Content in YSO and RSO as a Function of the Initial Fresh Weight

The phenolic increase observed after baking, frying and grilling could be a consequence of water loss during cooking, which resulted in a surge of onion phenolic compounds, or to an easier extractability due to a matrix softening effect [11,12]. Therefore, to gain more information on the stability/release of phenolic compounds, the values were corrected for the weight loss due to the cooking procedure (cooking ratio as reported in Table 1).

**Figure 1:**
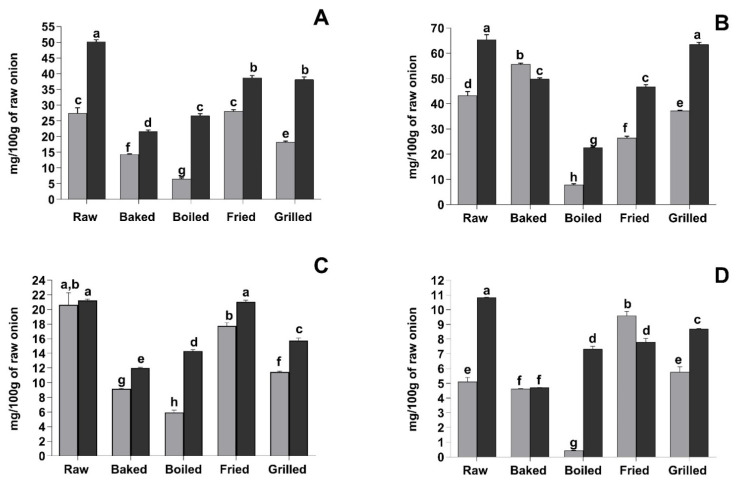
Effect of different cooking methods on YSO and RSO total phenolic compounds and quercetin-derivatives referred to the original fresh weight. Light grey bars indicated the YSO whereas black bars the RSO. (**A**) Total phenolic compounds measured by mass spectrometry. Results are expressed as mg/100 g of fresh weight. (**B**) Total phenolic compounds with the Folin-Ciocalteu’s assay. Results are expressed as mg of gallic acid equivalent/100 g of fresh weight. (**C**) Total quercetin-di-hexosides measured by mass spectrometry. Results are expressed as mg/100 g of fresh weight. (**D**) Total quercetin-mono-hexosides measured by mass spectrometry. Results are expressed as mg/100 g of fresh weight. Different letters mean significant difference (*p* < 0.05).

As reported in Figure 1A, all the treatments caused a significant reduction in extractable total phenolic compounds (sum of the amounts of individual phenolic compounds identified by mass spectrometry). The only exception was noted for fried YSO, where the amount of extractable total phenolic compounds was not significantly different (*p* > 0.05) compared to the raw sample. The highest loss of total phenolic compounds was recorded after boiling (76.2% loss) in YSO and after baking (56.9% loss) in RSO. In this context, the data acquired by mass spectrometry were fairly different compared to those observed with the Folin-Ciocalteu’s assay (Figure 1B). This incongruity may be due to the formation of Folin-Ciocalteu-reactive Maillard reaction products during cooking and/or to the thermally-induced degradation of Folin-Ciocalteu-reactive compounds (i.e., ascorbic acid or sulphur-containing compounds) [36,37,38].

The behaviour of quercetin-di-hexosides was similar between the two varieties (Figure 1C). Frying was the thermal treatment that caused the lowest decrease of quercetin-di-hexosides, whereas the highest losses were recorded after boiling and grilling. Considering quercetin-mono-hexosides, decreases of 56.4%, 32.3%, 27.9% and 19.7% were observed for baking, boiling, frying and grilling treatments in RSO, respectively, compared to the raw sample (Figure 1D). Contrariwise, grilling and, especially, frying boosted quercetin-mono-hexosides concentration in YSO (Figure 1D).

**Figure 2:**
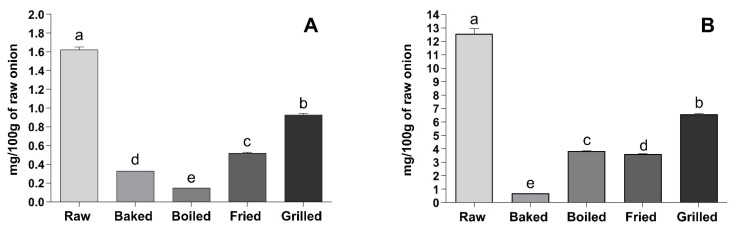
Effect of different cooking methods on YSO hydroxycinnamic acids and RSO anthocyanins referred to the original fresh weight. (**A**) Total hydroxycinnamic acids measured by mass spectrometry in YSO. Results are expressed as mg/100 g of fresh weight. (**B**) Total anthocyanins measured by mass spectrometry in RSO. Results are expressed as mg/100 g of fresh weight. Different letters indicate that the values are significantly different (*p* < 0.05).

Total hydroxycinnamic in the YSO and total anthocyanins in the RSO followed a similar trend resulting in a decrease for all of the thermal treatments (Figure 2A,B).

**Figure 3:**
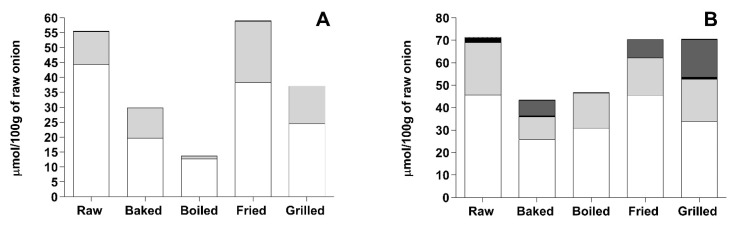
Sum of the molar concentrations of quercetin-derivatives and the degradation product protocatechuic acid-hexoside in YSO and RSO as affected by the cooking method. The data are referred to the original fresh weight and expressed as μmol of compound/100 g of fresh weight. (**A**) YSO; (**B**) RSO. Reported compounds are total quercetin-di-hexosides (white), total quercetin-mono-hexosides (light grey), quercetin (black) and protocatechuic acid-hexoside (dark grey).

In YSO, the molar ratio between quercetin-di-hexosides and quercetin-mono-hexosides decreased after baking, grilling and frying. The raw YSO had a quercetin-di-hexosides/quercetin-mono-hexosides molar ratio of 4.03 whereas baked, grilled and fried samples showed ratios of 1.98, 1.99 and 1.85, respectively. These results suggested that quercetin-di-hexosides were hydrolysed in the corresponding mono-hexosides during thermal treatments as already reported in other studies for YSO varieties [39]. This is particularly evident in fried YSO where the sum of the molar concentrations of the quercetin-derivatives was not significantly different respect to the raw sample (Figure 3A). During frying, quercetin-derivatives were quite stable and only a net molar conversion of quercetin-di-hexosides (≈8 μmol/100 g decrease respect to the raw sample) in quercetin-mono-hexosides (≈9 μmol/100 g increase respect to the raw sample) was observed. In particular, quercetin-3-*O*-hexoside-4′-*O*-hexoside was de-glycosylated to quercetin-4′-*O*-hexoside without any formation of the 3-*O*-hexoside (Table 2). As stated by Rohn et al. [39], the hexoside in 4′-*O*-position displayed a higher stability versus de-glycosylation than the hexoside in 3-*O*-position. However, contrary to the above-mentioned study, no increase in quercetin aglycone was found after frying. Similarly, also grilling and baking resulted in a decrease in the quercetin-di-hexosides/quercetin-mono-hexosides molar ratio. This aspect highlighted that the conversion of quercetin-3-*O*-hexoside-4′-*O*-hexoside in quercetin-4′-*O*-hexoside took place during these thermal treatments, without any formation of quercetin aglycone (Figure 3A and Table 2). On molar basis, boiling was the thermal treatment that triggered the highest loss of quercetin-derivatives, respect to the raw sample (75% loss) as a consequence of the leaching of these compounds in the cooking water as previously suggested [18,19,21].

In RSO, all the treatments caused a decrease in the sum of the molar concentrations of quercetin-derivatives, respect to the raw sample (Figure 3B). The highest decrease was observed after baking (49%), whereas the lowest one was detected after frying (13%). Furthermore, despite a decrease in the quercetin-di-hexosides amount (Table 3), the quercetin-di-hexosides/quercetin-mono-hexosides molar ratio after the thermal treatments was similar or higher than the raw sample. Altogether, these data suggest that both the quercetin-glycosides (mono- and di-hexosides) degraded in baked, grilled and fried samples without the occurrence of quercetin aglycone. Interestingly, as reported in Table 3, protocatechuic acid-hexoside, which was not present in the raw RSO, was first detected after baking, grilling and frying. Protocatechuic acid is a well-known quercetin degradation product, resulting from the oxidative decarboxylation by means of oxygen nucleophilic attack, followed by the cleavage of the C-ring [40,41]. We hypothesize that the same mechanism may occur in the case of quercetin-4′-*O*-hexoside resulting in the release of protocatechuic acid-hexoside in the food matrix. When the molar concentration of protocatechuic acid-hexoside was added to the sum of molar concentrations of quercetin-derivatives, a 100% recovery was observed after frying and grilling respect to the raw onion (Figure 3B).

**Figure 4:**
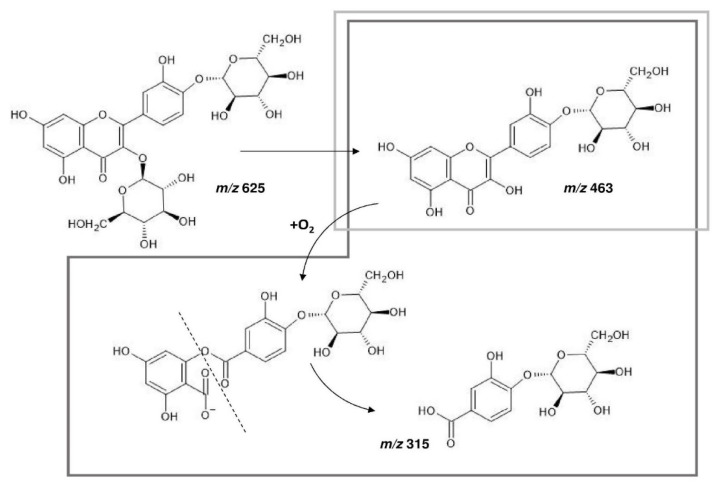
Proposed degradation pathway for quercetin-3-*O*-hexoside-4′-*O*-hexoside during cooking of YSO and RSO. Quercetin-3-*O*-hexoside-4′-*O*-hexoside (*m/z* = 625) was firstly de-glycosylated at the C3 level, releasing quercetin-4′-*O*-hexoside (*m/z* = 463), under heating treatment and in presence of oxygen. Quercetin-4′-*O*-hexoside may face the nucleophilic attack by oxygen and the cleavage of the C-ring, resulting in the release of protocatechuic acid-hexoside (*m/z* = 315). Light grey box records the hypothesized degradation pathway detected in YSO. Dark grey box records the hypothesized degradation pathway detected in RSO.

Furthermore, the molar ratio calculated by including the molar concentration of protocatechuic acid-hexoside at the denominator (i.e., quercetin-di-hexosides/(quercetin-mono-hexosides + protocatechuic acid-hexoside) molar ratio) gave values for frying (1.82 molar ratio) and grilling (0.96 molar ratio) significantly lower respect to the raw onion (1.95 molar ratio). In light of the degradation pathway proposed in Figure 4, these results suggest a net conversion of quercetin-3-*O*-hexoside-4′-*O*-hexoside in quercetin-4′-*O*-hexoside, which was further degraded in protocatechuic acid-hexoside.

Also during baking, the same hypothesized degradation pathway may occur, as suggested by the presence of protocatechuic acid-hexoside and the calculation of a molar ratio, which included the molar concentration of protocatechuic acid-hexoside at the denominator, lower than the raw sample (1.50 vs 1.95 molar ratio). However, the sum of the molar concentrations of the quercetin-derivatives and protocatechuic acid-hexoside accounted for only the 61% of the total amount of quercetin-derivatives in the raw onion. Similarly to YSO, boiled RSO confirmed lower quercetin-derivatives concentration than the raw sample, due to the leaching of these compounds in cooking water.

### 3.4. Effect of Cooking Methods on the Release and Bioaccessibility of YSO and RSO Phenolic Compounds

Recently, several works focused on the influence of domestic cooking processes on the release and the bioaccessibility of phenolic compounds [13,42,43,44,45,46]. Notwithstanding, the very high incidence of phenolic compounds in onion, no studies paid attention to characterize the possible correlation between the cooking procedures and the bioaccessibility of onion phenolic compounds after in vitro gastro-intestinal digestion.

Data reported in Table 4 and Table 5 show that the bioaccessibility index (BI) was below 100% for either raw or cooked samples in both the onion varieties.

In YSO, the BI for total phenolic compounds determined by mass spectrometry was between 42.6% and 65.5% in grilled and baked samples, respectively (Table 4).

Boiled YSO showed the lowest quantity of bioaccessible phenolic compounds (8.76 mg/100 g of cooked sample), since only quercetin-3-*O*-hexoside-4′-*O*-hexoside was released after the in vitro gastro-intestinal digestion. Baked YSO had the largest amount of total phenolic compounds after in vitro digestion (28.53 mg/100 g of cooked sample), followed by fried (22.12 mg/100 g of cooked sample) and grilled (16.50 mg/100 g of cooked sample) samples (Table 4). Raw YSO showed about half the quantity of bioaccessible total phenolic compounds of the baked sample. Total flavonols accounted for the 100% of bioaccessible phenolic compounds in all of the samples, with the exception of baked YSO, where only a mere 3% of hydroxycinnamic acids were detected.

Quercetin-3-*O*-hexoside-4′-*O*-hexoside was the most abundant phenolic compound in all of the yellow-skinned samples even though its BI was between 50.9% and 64.4% in grilled and baked onion, respectively. The second most abundant compound in all of the samples was quercetin-4′-*O*-hexoside, except for the boiled sample where it was not detected after in vitro digestion.

The highest amount of quercetin-3-*O*-hexoside-4′-*O*-hexoside and quercetin-4′-*O*-hexoside was found after in vitro digestion of baked YSO (Table 4).

In RSO, the BI for total phenolic compounds after in vitro gastro-intestinal digestion was between 39.8% and 80.2% in boiled and baked samples, respectively (Table 5). The boiled RSO sample also showed the lowest amount of total phenolic compounds after digestion (17.22 mg/100 g of cooked sample) whereas baked onion had the highest amount of bioaccessible phenolic compounds (45.19 mg/100 g of cooked sample). Quercetin-3-*O*-hexoside-4′-*O*-hexoside and quercetin-4′-*O*-hexoside were the most abundant bioaccessible phenolic compounds in all the RSO samples. The highest amount of quercetin-3-*O*-hexoside-4′-*O*-hexoside was detected after in vitro digestion of baked RSO, whereas grilled RSO showed the highest amount of quercetin-4′-*O*-hexoside (Table 5). Total flavonols accounted for more than 95% of total bioaccessible phenolic compounds in all the RSO samples. Anthocyanins showed low bioaccessibility (between 3.8% and 17.6%) and were not detected after digestion of the baked sample. The highest amount of bioaccessible anthocyanins was found in fried RSO, but they accounted only for the 3.2% of total bioaccessible phenolic compounds (Table 5).

Generally, the BI calculated for the total phenolic compounds through the Folin-Ciocalteu’s assay was higher than that calculated by mass spectrometry for both the onion varieties (Table 4 and Table 5). In most cases, the BI was greater than 100% suggesting the presence of gastro-intestinal interfering products, which were actually absent during the extraction with the methanol/formic acid solution.

### 3.5. Effect of Cooking and In Vitro Gastro-Intestinal Digestion on the Antioxidant Activity of YSO and RSO

**Figure 5:**
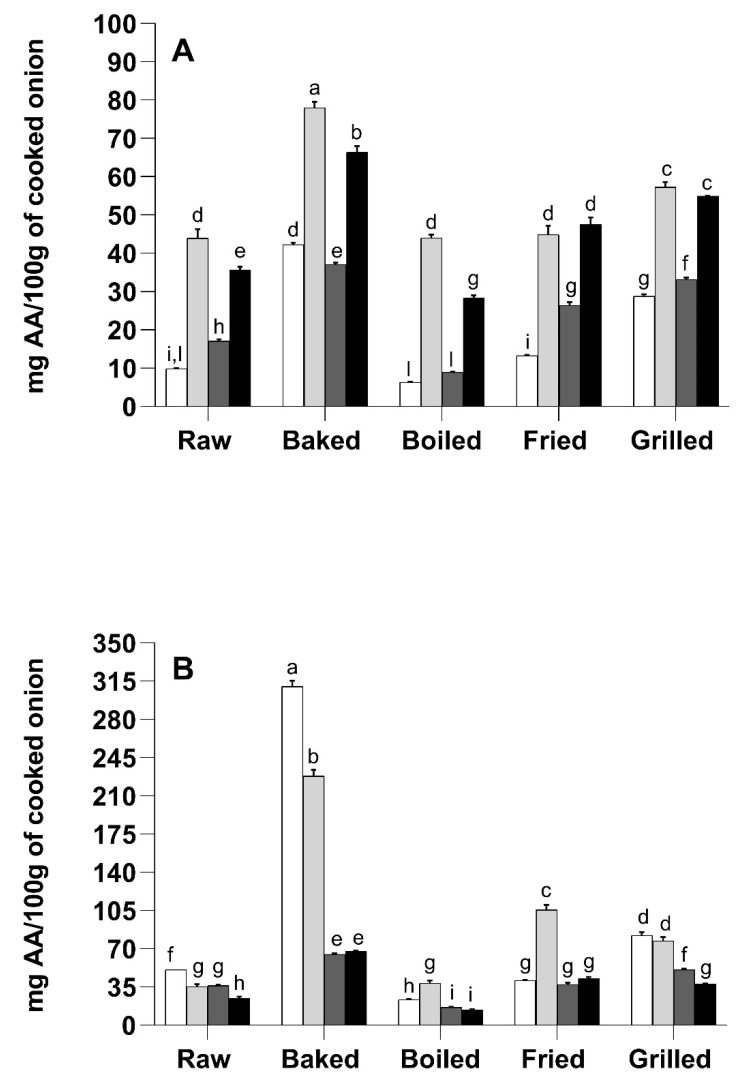
Effect of different cooking methods and in vitro digestion on yellow-skinned (YSO) and red-skinned (RSO) onions antioxidant activity. (**A**) YSO antioxidant activity as determined by the ABTS assay in the methanolic/formic acid extract (white) and after in vitro digestion (light grey bars). RSO antioxidant activity as determined by the ABTS assay in the methanolic/formic acid extract (dark grey bars) and after in vitro digestion (black bars). (**B**) YSO antioxidant activity as determined by the FRAP assay in the methanolic/formic acid extract (light grey bars) and after in vitro digestion (grey bars). RSO antioxidant activity as determined by the FRAP assay in the methanolic/formic acid extract (dark grey bars) and after in vitro digestion (black bars). Results are expressed as mg of ascorbic acid equivalent/100 g of raw or cooked onion. Different letters indicate that the values are significantly different (*p* < 0.05).

Figure 5 depicts the impact of cooking methods and in vitro digestion on the antioxidant activity of YSO and RSO phenolic compounds, tested with the ABTS and FRAP assays. Generally, the ABTS and FRAP data showed a significant correlation with the total phenolic compounds determined with the Folin-Ciocalteu assay (Pearson coefficient *r =* 0.968 *p =* 4.3 × 10^−6^ and Pearson coefficient *r =* 0.752 *p =* 0.012, for ABTS and FRAP assay, respectively). On the contrary, no significant correlations were found between the antioxidant activities data and the total amount of phenolic compounds determined by mass spectrometry.

The highest ABTS and FRAP values were found for baked samples, whereas the lowest values were found for boiled samples in both the onion varieties (Figure 5). In the ABTS assay, the raw YSO and RSO exhibited higher antioxidant activity than the boiled samples but lower than the baked, grilled and fried ones. In the FRAP assay, baking and grilling resulted in an increased antioxidant activity respect to the raw samples whereas boiling determined a decrease in the FRAP values in both the onion varieties. In YSO, frying also caused a decrease in the FRAP value respect to the raw sample, whereas in the RSO no significant differences were found between fried and raw onion.

Conflicting results are present in literature about the variation in the antioxidant activity after onion cooking. According to the present study, Pellegrini et al. [47] found an increase in the antioxidant activity determined with the ABTS assay after frying respect to the raw onion. However, other authors found no loss or decrease in the ABTS values after grilling and frying [16,48].

After in vitro digestion, baked samples still showed the highest ABTS and FRAP values in both the onion varieties (Figure 5). In the ABTS assay, raw and cooked YSO and RSO showed higher antioxidant activity after digestion compared to the methanol/formic acid extract, suggesting that the digestion procedure released antioxidant compounds, which were not extracted with the methanol/formic acid solution. On the contrary, when antioxidant activity was assayed with the FRAP procedure, only frying, for both the onion varieties, and boiling for YSO showed higher data after in vitro digestion respect to the methanol/formic acid extracts.

## 4. Conclusions

This study demonstrates that cooking treatments may modulate the release and bioaccessibility of onion phenolic compounds. Based on the initial fresh weight, all the treatments tended to decrease the phenolic compounds content respect to the raw samples as determined by mass spectrometry. The only exception was found for fried YSO. However, when the results were expressed referring to 100 g of cooked samples, baking, frying and grilling resulted in an increased amount of phenolic compounds in comparison with the raw onion samples. After in vitro digestion, baking for both the onion varieties and grilling for RSO significantly increased the bioaccessibility of onion phenolic compounds, fostering their delivery from the onion matrices. For both the onion varieties, baking was the thermal treatment that provided the highest amount of phenolic compounds after in vitro digestion. In particular, baking and grilling resulted in an increased gastro-intestinal availability of quercetin-3-*O*-hexoside-4′-*O*-hexoside, underlining the highest bioaccessibility respect to its aglycone or mono-hexosides. Considering its reported anti-proliferative activity against colon cancer cell lines, it is a potential front-runner in the prevention strategies designing [49,50]. Additionally, quercetin-3-*O*-hexoside-4′-*O*-hexoside can also be absorbed due to its considerable hydrophilic character and undergo further de-glycosylation at intestinal level. The corresponding aglycone may actually be absorbed and reach the systemic level where it can carry out its biological actions [50].

These results suggest how baking and grilling are the recommended cooking methods, not only for the healthy lack of use of cooking oils or fats (i.e., sunflower oil in frying), but also for the evidence of the proved bioaccessibility characteristics of onion health-promoting phenolic compounds (e.g., flavonols).

## Figures and Tables

**Table 1:** Cooking parameters and weight loss during cooking of yellow-skinned (YSO) and red-skinned onion (RSO).

	YSO				RSO			
	Baking	Boiling	Frying	Grilling	Baking	Boiling	Frying	Grilling
Cooking temperature (°C)	180	100	140	110	180	100	140	110
Cooking time (min)	30	30	8	15	30	30	8	15
Initial weight (g)	173	129	200	170	133	129	158	155
Final weight (g)	57	49	126	80	48	79	90	88
Weight loss (%)	67	62	37	53	64	38	43	43
Initial/final weight ratio	3.04	2.63	1.59	2.13	2.77	1.63	1.76	1.76

**Table 2:** Amount of phenolic compounds in raw and cooked yellow-skinned onion (YSO). Results are expressed in mg of phenolic compound/100 g of raw or cooked onion.

	YSO				
Compound	Raw	Baked	Boiled	Fried	Grilled
	Hydroxycinnamic acids				
Caffeic acid-*O*-hexoside	0.41 ± 0.01 ^a^	n.d.	n.d.	n.d.	0.03 ± 0.00 ^b^
Ferulic acid-*O*-hexoside	0.43 ± 0.02 ^a^	n.d.	n.d.	n.d.	0.10 ± 0.01 ^b^
Sinapic acid-*O*-hexoside isomer	0.11 ± 0.00 ^c^	n.d.	0.08 ± 0.00 ^d^	0.25 ± 0.00 ^b^	0.46 ± 0.00 ^a^
Sinapic acid-*O*-hexoside isomer	0.66 ± 0.01 ^c^	0.99 ± 0.01 ^b^	0.30 ± 0.00 ^e^	0.58 ± 0.01 ^d^	1.39 ± 0.03 ^a^
Total hydroxycinnamic acids	1.61 ± 0.03 ^b^	0.99 ± 0.01 ^c^	0.38 ± 0.01 ^e^	0.83 ± 0.01 ^d^	1.98 ± 0.03 ^a^
	Flavonols				
Quercetin	0.03 ± 0.00 ^a^	n.d.	n.d.	0.01 ± 0.00 ^b^	0.03 ± 0.00 ^a^
Quercetin-3-*O*-hexoside	0.07 ± 0.00 ^b^	0.06 ± 0.00 ^b^	0.03 ± 0.00 ^c^	0.13 ± 0.00 ^a^	n.d.
Quercetin-4′-*O*-hexoside	4.99 ± 0.29 ^c^	13.76 ± 0.06 ^a,b^	1.00 ± 0.05 ^d^	15.01 ± 0.48 ^a^	12.07 ± 0.77 ^b^
Quercetin-3-*O*-glucoside	n.d.	0.19 ± 0.01 ^a^	0.04 ± 0.00 ^c^	n.d.	0.12 ± 0.00 ^b^
Quercetin-3-*O*-hexoside-7-*O*-hexoside	0.02 ± 0.00	n.d.	n.d.	n.d.	n.d.
Quercetin-7-*O*-hexoside-4′-*O*-hexoside	0.04 ± 0.00 ^b^	0.13 ± 0.00 ^a^	n.d.	n.d.	0.05 ± 0.00 ^b^
Quercetin-3-*O*-hexoside-4′-*O*-hexoside	20.56 ± 1.68 ^c^	27.67 ± 0.21 ^a^	15.58 ± 0.98 ^d^	28.05 ± 0.66 ^a^	24.26 ± 0.27 ^b^
Quercetin-tri-*O*-hexoside	0.02 ± 0.00 ^b^	0.06 ± 0.00 ^a^	0.02 ± 0.00 ^b^	0.03 ± 0.00 ^b^	n.d.
Isorhamnetin-4′-*O*-hexoside	0.06 ± 0.00 ^d^	0.45 ± 0.00 ^a^	0.05 ± 0.00 ^d^	0.19 ± 0.00 ^b^	0.15 ± 0.00 ^c^
Isorhamnetin-3-*O*-hexoside-4′-*O*-hexoside	0.02 ± 0.00 ^d^	0.21 ± 0.01 ^a^	0.05 ± 0.00 ^c^	0.06 ± 0.00 ^b,c^	0.07 ± 0.00 ^b^
Total flavonols	25.81 ± 1.70 ^c^	42.53 ± 0.22 ^a^	16.77 ± 0.98 ^d^	43.48 ± 0.81 ^a^	36.75 ± 0.82 ^b^
					
Total phenolic by MS *	27.42 ± 1.70 ^c^	43.52 ± 0.22 ^a^	17.15 ± 0.98 ^d^	44.31 ± 0.81 ^a^	38.73 ± 0.82 ^b^
					
Total phenolic by FC **	43.21 ± 1.57 ^d^	168.75 ± 1.03 ^a^	20.85 ± 0.85 ^e^	51.71 ± 1.13 ^c^	79.11 ± 0.31 ^b^

Different letters within the same row mean significant different (*p* < 0.05) values. n.d. means that the compound was not detected in the sample. * Total phenolic compounds by mass spectrometry. Sum of the amount of individual phenolic compounds. ** Total phenolic compounds by Folin-Ciocalteu’s assay. Data are expressed as mg of gallic acid equivalent per 100 g of raw or cooked onion.

**Table 3:** Amount of phenolic compounds in raw and cooked red-skinned onion (RSO). Results are expressed in mg of phenolic compound/100 g of raw or cooked onion.

	RSO				
Compound	Raw	Baked	Boiled	Fried	Grilled
	Hydroxybenzoic acids				
Protocatechuic acid-*O*-hexoside	n.d.	2.99 ± 0.03 ^b^	n.d.	2.20 ± 0.06 ^c^	4.56 ± 0.03 ^a^
Total hydroxybenzoic acids	n.d.	2.99 ± 0.03 ^b^	n.d.	2.20 ± 0.06 ^c^	4.56 ± 0.03 ^a^
	Flavan-3-ols				
(Epi)catechin-3-*O*-hexoside isomer	0.04 ± 0.00 ^d^	0.72 ± 0.01 ^b^	0.02 ± 0.00 ^e^	0.20 ± 0.00 ^c^	0.82 ± 0.02 ^a^
(Epi)catechin-3-*O*-hexoside isomer	0.01 ± 0.00	n.d.	n.d.	n.d.	n.d.
Total flavan-3-ols	0.05 ± 0.00 ^d^	0.72 ± 0.01 ^b^	0.02 ± 0.00 ^e^	0.20 ± 0.00 ^c^	0.82 ± 0.02 ^a^
	Di-hydro-flavonols				
Taxifolin-*O*-hexoside isomer	0.06 ± 0.00 ^b^	n.d.	0.04 ± 0.00 ^b^	0.06 ± 0.00 ^b^	0.10 ± 0.00 ^a^
Taxifolin-*O*-hexoside isomer	0.06 ± 0.00 ^d^	n.d.	0.20 ± 0.00 ^b^	0.29 ± 0.00 ^a^	0.16 ± 0.00 ^c^
Taxifolin-*O*-hexoside isomer	0.05 ± 0.00 ^b^	0.09 ± 0.00a	n.d.	0.04 ± 0.00 ^b^	0.02 ± 0.00 ^c^
Taxifolin-*O*-hexoside isomer	0.12 ± 0.00 ^a^	0.06 ± 0.00 ^c^	0.03 ± 0.00 ^d^	0.09 ± 0.00 ^b^	0.05 ± 0.00 ^c^
Taxifolin-*O*-hexoside isomer	n.d.	n.d.	n.d.	0.07 ± 0.00	n.d.
Total di-hydro-flavonols	0.29 ± 0.00 ^c^	0.15 ± 0.00 ^d^	0.27 ± 0.00 ^c^	0.55 ± 0.01 ^a^	0.33 ± 0.00 ^b^
	Flavonols				
Quercetin	0.89 ± 0.05 ^a^	0.43 ± 0.00 ^b^	0.06 ± 0.01 ^c^	0.03 ± 0.01 ^c^	0.73 ± 0.01 ^a^
Quercetin-3-*O*-hexoside	0.36 ± 0.01 ^d^	1.01 ± 0.01 ^a^	0.29 ± 0.00 ^d^	0.71 ± 0.01 ^b^	0.58 ± 0.00 ^c^
Quercetin-4′-*O*-hexoside	10.46 ± 0.01 ^d^	12.08 ± 0.05 ^b,c^	11.47 ± 0.31 ^c^	12.94 ± 0.46 ^a,b^	13.49 ± 0.06 ^a^
Quercetin-3-*O*-hexoside-7-*O*-hexoside	n.d.	n.d.	n.d.	n.d.	0.15 ± 0.00
Quercetin-7-*O*-hexoside-4′-*O*-hexoside	0.37 ± 0.00 ^c^	0.65 ± 0.00 ^a^	0.31 ± 0.00 ^d^	0.55 ± 0.00 ^b^	0.38 ± 0.00 ^c^
Quercetin-3-*O*-hexoside-4′-*O*-hexoside	20.87 ± 0.14 ^c^	32.55 ± 0.30 ^a^	23.02 ± 0.34 ^c^	36.29 ± 0.37 ^a^	27.20 ± 0.58 ^b^
Quercetin-tri-*O*-hexoside isomer	0.15 ± 0.00 ^a^	0.14 ± 0.00 ^a^	0.11 ± 0.00 ^b^	n.d.	0.14 ± 0.00 ^a^
Quercetin-tri-*O*-hexoside isomer	n.d.	0.03 ± 0.00 ^a^	n.d.	0.02 ± 0.00 ^a^	0.02 ± 0.00 ^a^
Kaempferol-7-*O*-hexoside isomer	0.26 ± 0.01 ^b^	0.05 ± 0.00 ^d^	0.20 ± 0.00 ^c^	0.20 ± 0.00 ^c^	0.33 ± 0.00 ^a^
Kaempferol-7-*O*-hexoside isomer	n.d.	0.07 ± 0.00 ^a^	0.02 ± 0.00 ^c^	0.02 ± 0.00 ^c^	0.04 ± 0.00 ^b^
Kaempferol-3-*O*-hexoside isomer	n.d.	0.09 ± 0.00 ^a^	n.d.	0.02 ± 0.00 ^b^	0.01 ± 0.00 ^b^
Kaempferol-3-*O*-hexoside-7-*O*-hexoside isómer	0.55 ± 0.00 ^a^	0.19 ± 0.00 ^b^	0.04 ± 0.00 ^d^	0.32 ± 0.00 ^c^	n.d.
Kaempferol-3-*O*-hexoside-7-*O*-hexoside isómer	0.07 ± 0.00 ^b^	0.16 ± 0.00 ^a^	0.05 ± 0.00 ^b^	0.08 ± 0.00 ^b^	n.d.
Kaempferol-hexoside-rhamnoside-rhamnoside	0.05 ± 0.00 ^a^	0.04 ± 0.00 ^a^	n.d.	n.d.	n.d.
Isorhamnetin-3-*O*-hexoside isomer	0.28 ± 0.00 ^c^	0.98 ± 0.00 ^b^	n.d.	1.23 ± 0.02 ^a^	0.81 ± 0.01 ^b^
Isorhamnetin-3-*O*-hexoside isomer	n.d.	0.21 ± 0.00 ^a^	n.d.	0.12 ± 0.01 ^b^	0.13 ± 0.01 ^b^
Isorhamnetin-4′-*O*-hexoside	2.25 ± 0.04 ^d^	2.82 ± 0.04 ^b^	n.d.	3.55 ± 0.07 ^a^	2.47 ± 0.01 ^c^
Isorhamnetin-3-*O*-hexoside-4′-*O*-hexoside	0.52 ± 0.00 ^e^	2.38 ± 0.03 ^a^	1.05 ± 0.05 ^d^	2.20 ± 0.01 ^b^	1.40 ± 0.01 ^c^
Isorhamnetin-*O*-hexoside-*O*-pentoside	0.11 ± 0.00 ^a^	n.d.	n.d.	0.04 ± 0.00 ^b^	n.d.
Myricetin-*O*-hexoside-*O*-hexoside isómer	n.d.	0.11 ± 0.00 ^a^	0.04 ± 0.00 ^c^	0.04 ± 0.00 ^c^	0.07 ± 0.00 ^b^
Myricetin-*O*-hexoside-*O*-hexoside isómer	n.d.	n.d.	n.d.	0.04 ± 0.00	n.d.
Total flavonols	37.19 ± 0.53 ^c^	53.98 ± 0.78 ^a^	36.66 ± 0.88 ^c^	58.40 ± 0.99 ^a^	47.95 ± 1.08 ^b^
	Anthocyanins				
Cyanidin-3-*O*-hexoside	1.00 ± 0.02 ^a^	0.36 ± 0.01 ^c^	0.74 ± 0.01 ^b^	n.d.	n.d.
Cyanidin-*O*-hexoside-*O*-hexoside isómer	0.09 ± 0.00 ^b^	n.d.	0.07 ± 0.00 ^b^	0.07 ± 0.00 ^b^	0.31 ± 0.02 ^a^
Cyanidin-*O*-hexoside-*O*-hexoside isómer	0.31 ± 0.01 ^b^	0.10 ± 0.00 ^d^	0.14 ± 0.00 ^c^	0.07 ± 0.01 ^d^	2.20 ± 0.02 ^a^
Cyanidin-*O*-malonyl-hexoside isomer	5.97 ± 0.37 ^a^	n.d.	3.25 ± 0.03 ^b^	2.48 ± 0.01 ^c^	4.93 ± 0.08 ^a^
Cyanidin-*O*-malonyl-hexoside isomer	n.d.	n.d.	n.d.	0.50 ± 0.01	n.d.
Cyanidin-*O*-hexoside-*O*-malonyl-hexoside isómer	4.60 ± 0.01 ^a^	1.29 ± 0.07 ^d^	1.67 ± 0.03 ^c^	2.82 ± 0.06 ^b^	3.96 ± 0.08 ^a^
Peonidin-3-*O*-hexoside	0.21 ± 0.00 ^a^	n.d.	0.13 ± 0.00 ^b^	n.d.	0.16 ± 0.00 ^b^
Peonidin-*O*-malonyl-hexoside	0.37 ± 0.01 ^a^	0.17 ± 0.00 ^b^	0.19 ± 0.00 ^b^	0.39 ± 0.00 ^a^	n.d.
Malvidin-*O*-hexoside-acetaldehyde	0.02 ± 0.00 ^a^	n.d.	0.02 ± 0.00 ^a^	n.d.	n.d.
Total anthocyanins	12.57 ± 0.38 ^a^	1.92 ± 0.07 ^c^	6.22 ± 0.04 ^b^	6.33 ± 0.06 ^b^	11.57 ± 0.12 ^a^
Total phenolic by MS *	50.12 ± 0.65 ^c^	59.76 ± 0.78 ^b^	43.15 ± 0.89 ^d^	67.70 ± 0.99 ^a^	65.22 ± 1.09 ^a^
Total phenolic by FC **	65.44 ± 1.88 ^d^	137.85 ± 1.23 ^a^	36.79 ± 0.31 ^e^	81.85 ± 0.54 ^c^	111.79 ± 1.44 ^b^

Different letters within the same row mean significant different (*p* < 0.05) values. n.d. means that the compound was not detected in the sample. * Total phenolic compounds by mass spectrometry. Sum of the amount of individual phenolic compounds. ** Total phenolic compounds by Folin-Ciocalteu’s assay. Data are expressed as mg of gallic acid equivalent per 100 g of raw or cooked onion.

**Table 4:** Amount of phenolic compounds in raw and cooked yellow-skinned onion (YSO) after in vitro gastro-intestinal digestion. Results are expressed in mg of phenolic compound/100 of raw or cooked onion. Bioaccessibility index (BI) is the percentage ratio between the concentration after in vitro gastro-intestinal digestion and the concentration before digestion.

	YSO									
Compound	Raw		Baked		Boiled		Fried		Grilled	
	After Digestion	BI (%)	After Digestion	BI (%)	After Digestion	BI (%)	After Digestion	BI (%)	After Digestion	BI (%)
Hydroxycinnamic acids										
Caffeic acid-*O*-hexoside	n.d.	n.d.	n.d.	n.d.	n.d.	n.d.	n.d.	n.d.	n.d.	n.d.
Ferulic acid-*O*-hexoside	n.d.	n.d.	n.d.	n.d.	n.d.	n.d.	n.d.	n.d.	n.d.	n.d.
Sinapic acid-*O*-hexoside isómer	n.d.	n.d.	n.d.	n.d.	n.d.	n.d.	n.d.	n.d.	n.d.	n.d.
Sinapic acid-*O*-hexoside isómer	n.d.	n.d.	0.76 ± 0.01	77.1	n.d.	n.d.	n.d.	n.d.	n.d.	n.d.
Total hydroxycinnamic acids	n.d.	n.d.	0.76 ± 0.01	77.1	n.d.	n.d.	n.d.	n.d.	n.d.	n.d.
Flavonols										
Quercetin	n.d.	n.d.	n.d.	n.d.	n.d.	n.d.	n.d.	n.d.	n.d.	n.d.
Quercetin-3-*O*-hexoside	0.02 ± 0.00 ^c^	29.3	0.19 ± 0.00 ^a^	264.1	n.d.	n.d.	0.10 ± 0.00 ^b^	80.3	0.09 ± 0.00 ^b^	n.d.
Quercetin-4′-*O*-hexoside	3.20 ± 0.00 ^d^	64.0	9.17 ± 0.16 ^a^	66.6	n.d.	n.d.	5.67 ± 0.18 ^b^	37.8	4.01 ± 0.02 ^c^	33.2
Quercetin-3-*O*-glucoside	n.d.	n.d.	n.d.	n.d.	n.d.	n.d.	n.d.	n.d.	n.d.	n.d.
Quercetin-3-*O*-hexoside-7-*O*-hexoside	n.d.	n.d.	n.d.	n.d.	n.d.	n.d.	n.d.	n.d.	n.d.	n.d.
Quercetin-7-*O*-hexoside-4′-*O*-hexoside	n.d.	n.d.	0.14 ± 0.00	112.9	n.d.	n.d.	n.d.	n.d.	n.d.	n.d.
Quercetin-3-*O*-hexoside-4′-*O*-hexoside	11.54 ± 0.13 ^b^	56.1	17.83 ± 1.09 ^a^	64.4	8.76 ± 0.12 ^c^	56.2	16.24 ± 0.18 ^a^	57.9	12.35 ± 0.49 ^b^	50.9
Quercetin-tri-*O*-hexoside	n.d.	n.d.	0.06 ± 0.00	86.3	n.d.	n.d.	n.d.	n.d.	n.d.	n.d.
Isorhamnetin-4′-*O*-hexoside	0.02 ± 0.00 ^c^	38.8	0.19 ± 0.00 ^a^	43.3	n.d.	n.d.	0.06 ± 0.00 ^b^	30.4	n.d.	n.d.
Isorhamnetin-3-*O*-hexoside-4′-*O*-hexoside	n.d.	n.d.	0.19 ± 0.00 ^a^	92.0	n.d.	n.d.	0.04 ± 0.00 ^b^	74.2	0.05 ± 0.00 ^b^	65.9
Total flavonols	14.78 ± 0.13 ^d^	57.2	27.77 ± 1.11 ^a^	65.3	8.76 ± 0.12 ^e^	52.2	22.12 ± 0.26 ^b^	50.9	16.50 ± 0.49 ^c^	44.9
Total phenolic by MS *	14.78 ± 0.13 ^d^	53.8	28.53 ± 1.11 ^a^	65.5	8.76 ± 0.12 ^e^	51.0	22.12 ± 0.26 ^b^	49.9	16.50 ± 0.49 ^c^	42.6
Total phenolic by FC **	63.38 ± 3.13 ^c^	146.7	207.66 ± 7.08 ^a^	123.1	57.24 ± 2.19 ^d^	117.8	74.76 ± 4.14 ^c^	179.2	111.23 ± 0.43 ^b^	140.6

Different letters within the same row mean significant different (*p* < 0.05) values. n.d. means that the compound was not detected in the sample. * Total phenolic compounds by mass spectrometry. Sum of the amount of individual phenolic compounds. ** Total phenolic compounds by Folin-Ciocalteu’s assay. Data are expressed as mg of gallic acid equivalent per 100 g of raw or cooked onion.

**Table 5:** Amount of phenolic compounds in raw and cooked red-skinned onion (RSO) after in vitro gastro-intestinal digestion. Results are expressed in mg of phenolic compound/100 of raw or cooked onion. Bioaccessibility index (BI) is the percentage ratio between the concentration after in vitro gastro-intestinal digestion and the concentration before digestion.

	RSO									
Compound	Raw		Baked		Boiled		Fried		Grilled	
	After Digestion	BI (%)	After Digestion	BI (%)	After Digestion	BI (%)	After Digestion	BI (%)	After Digestion	BI (%)
Hydroxybenzoic acids										
Protocatechuic acid-*O*-hexoside	n.d.	n.d.	n.d.	n.d.	n.d.	n.d.	n.d.	n.d.	n.d.	n.d.
Total hydroxybenzoic acids	n.d.	n.d.	n.d.	n.d.	n.d.	n.d.	n.d.	n.d.	n.d.	n.d.
Flavan-3-ols										
(Epi)catechin-3-*O*-hexoside isomer	n.d.	n.d.	0.51 ± 0.00 ^b^	70.0	n.d.	n.d.	0.27 ± 0.01 ^c^	133.4	0.71 ± 0.10 ^a^	86.3
(Epi)catechin-3-*O*-hexoside isomer	n.d.	n.d.	n.d.	n.d.	n.d.	n.d.	n.d.	n.d.	n.d.	n.d.
Total flavan-3-ols	n.d.	n.d.	0.51 ± 0.00 ^b^	70.0	n.d.	n.d.	0.27 ± 0.01 ^c^	133.4	0.71 ± 0.10 ^a^	86.3
Di-hydro-flavonols										
Taxifolin-*O*-hexoside isomer	n.d.	n.d.	n.d.	n.d.	n.d.	n.d.	n.d.	n.d.	n.d.	n.d.
Taxifolin-*O*-hexoside isomer	n.d.	n.d.	n.d.	n.d.	n.d.	n.d.	n.d.	n.d.	n.d.	n.d.
Taxifolin-*O*-hexoside isomer	0.03 ± 0.00 ^b^	56.4	n.d.	n.d.	n.d.	n.d.	0.08 ± 0.00 ^a^	217.5	n.d.	n.d.
Taxifolin-*O*-hexoside isomer	0.03 ± 0.00	24.0	n.d.	n.d.	n.d.	n.d.	n.d.	n.d.	n.d.	n.d.
Taxifolin-*O*-hexoside isomer	n.d.	n.d.	n.d.	n.d.	n.d.	n.d.	n.d.	n.d.	n.d.	n.d.
Total di-hydro-flavonols	0.06 ± 0.00 ^b^	19.8	n.d.	n.d.	n.d.	n.d.	0.08 ± 0.00 ^a^	217.5	n.d.	n.d.
Flavonols										
Quercetin	n.d.	n.d.	n.d.	n.d.	n.d.	n.d.	n.d.	n.d.	n.d.	n.d.
Quercetin-3-*O*-hexoside	n.d.	n.d.	1.28 ± 0.01 ^a^	126.9	n.d.	n.d.	0.89 ± 0.09 ^b^	126.6	0.34 ± 0.01 ^c^	19.1
Quercetin-4′-*O*-hexoside	4.27 ± 0.01 ^a^	40.8	5.59 ± 0.06 ^b^	46.3	1.89 ± 0.03 ^c^	16.5	4.99 ± 0.05 ^d^	38.5	7.07 ± 0.06 ^e^	52.4
Quercetin-3-*O*-hexoside-7-*O*-hexoside	n.d.	n.d.	n.d.	n.d.	n.d.	n.d.	n.d.	n.d.	n.d.	n.d.
Quercetin-7-*O*-hexoside-4′-*O*-hexoside	0.22 ± 0.01 ^b^	59.7	0.47 ± 0.01 ^a^	72.6	n.d.	n.d.	0.20 ± 0.02 ^b^	36.6	0.26 ± 0.01 ^b^	68.0
Quercetin-3-*O*-hexoside-4′-*O*-hexoside	17.73 ± 0.17 ^d^	84.9	35.35 ± 0.36 ^a^	108.6	14.70 ± 0.73 ^e^	63.8	25.08 ± 0.22 ^c^	69.1	31.73 ± 0.52 ^b^	116.7
Quercetin-tri-*O*-hexoside isómer	0.11 ± 0.00 ^c^	73.0	0.23 ± 0.00 ^a^	165.6	0.11 ± 0.00 ^c^	102.3	0.14 ± 0.00 ^b^	n.d.	0.23 ± 0.00 ^a^	204.6
Quercetin-tri-*O*-hexoside isómer	n.d.	n.d.	0.09 ± 0.00	279.7	n.d.	n.d.	n.d.	n.d.	n.d.	n.d.
Kaempferol-7-*O*-hexoside isomer	0.05 ± 0.00	18.0	n.d.	n.d.	n.d.	n.d.	n.d.	n.d.	n.d.	n.d.
Kaempferol-7-*O*-hexoside isomer	n.d.	n.d.	n.d.	n.d.	n.d.	n.d.	n.d.	n.d.	n.d.	n.d.
Kaempferol-3-*O*-hexoside isomer	n.d.	n.d.	n.d.	n.d.	n.d.	n.d.	n.d.	n.d.	n.d.	n.d.
Kaempferol-3-*O*-hexoside-7-*O*-hexoside isomer	0.13 ± 0.00	23.2	n.d.	n.d.	n.d.	n.d.	n.d.	n.d.	n.d.	n.d.
Kaempferol-3-*O*-hexoside-7-*O*-hexoside isomer	n.d.	n.d.	n.d.	n.d.	n.d.	n.d.	n.d.	n.d.	n.d.	n.d.
Kaempferol-hexoside-rhamnoside-rhamnoside	n.d.	n.d.	n.d.	n.d.	n.d.	n.d.	n.d.	n.d.	n.d.	n.d.
Isorhamnetin-3-*O*-hexoside isomer	n.d.	n.d.	n.d.	n.d.	n.d.	n.d.	0.55 ± 0.01 ^b^	44.9	0.60 ± 0.00 ^a^	73.8
Isorhamnetin-3-*O*-hexoside isomer	n.d.	n.d.	n.d.	n.d.	n.d.	n.d.	n.d.	n.d.	n.d.	n.d.
Isorhamnetin-4′-*O*-hexoside	n.d.	n.d.	n.d.	n.d.	n.d.	n.d.	n.d.	n.d.	n.d.	n.d.
Isorhamnetin-3-*O*-hexoside-4′-*O*-hexoside	0.92 ± 0.01 ^b^	174.8	1.68 ± 0.02 ^a^	70.5	0.29 ± 0.00 ^d^	27.4	0.85 ± 0.02 ^c^	38.7	0.73 ± 0.08 ^c^	52.4
Isorhamnetin-*O*-hexoside-*O*-pentoside	0.08 ± 0.00 ^b^	71.6	n.d.	n.d.	n.d.	n.d.	0.14 ± 0.00 ^a^	344.8	n.d.	n.d.
Myricetin-di-*O*-hexoside	n.d.	n.d.	n.d.	n.d.	n.d.	n.d.	n.d.	n.d.	n.d.	n.d.
Myricetin-di-*O*-hexoside	n.d.	n.d.	n.d.	n.d.	n.d.	n.d.	n.d.	n.d.	n.d.	n.d.
Total flavonols	23.50 ± 0.17 ^d^	64.7	44.68 ± 0.37 ^a^	83.4	16.98 ± 0.73 ^e^	46.2	32.85 ± 0.25 ^c^	56.3	41.02 ± 0.53 ^b^	83.6
Anthocyanins										
Cyanidin-3-*O*-hexoside	n.d.	n.d.	n.d.	n.d.	n.d.	n.d.	n.d.	n.d.	0.11 ± 0.00	n.d.
Cyanidin-*O*-hexoside-*O*-hexoside isómer	n.d.	n.d.	n.d.	n.d.	n.d.	n.d.	n.d.	n.d.	n.d.	n.d.
Cyanidin-*O*-hexoside-*O*-hexoside isómer	n.d.	n.d.	n.d.	n.d.	n.d.	n.d.	0.24 ± 0.00	328.2	n.d.	n.d.
Cyanidin-*O*-malonyl-hexoside isomer	0.52 ± 0.00 ^b^	8.7	n.d.	n.d.	0.14 ± 0.00 ^d^	4.2	0.60 ± 0.00 ^a^	24.0	0.45 ± 0.00 ^c^	9.2
Cyanidin-*O*-malonyl-hexoside isomer	n.d.	n.d.	n.d.	n.d.	n.d.	n.d.	n.d.	n.d.	n.d.	n.d.
Cyanidin-*O*-hexoside-*O*-malonyl-hexoside isomer	0.31 ± 0.00 ^a^	6.7	n.d.	n.d.	0.10 ± 0.00 ^c^	5.8	0.28 ± 0.00 ^b^	9.9	0.34 ± 0.01 ^a^	8.4
Peonidin-3-*O*-hexoside	n.d.	n.d.	n.d.	n.d.	n.d.	n.d.	n.d.	n.d.	n.d.	n.d.
Peonidin-*O*-malonyl-hexoside	n.d.	n.d.	n.d.	n.d.	n.d.	n.d.	n.d.	n.d.	n.d.	n.d.
Malvidin-*O*-hexoside-acetaldehyde	n.d.	n.d.	n.d.	n.d.	n.d.	n.d.	n.d.	n.d.	n.d.	n.d.
Total anthocyanins	0.83 ± 0.00 ^c^	6.6	n.d.	n.d.	0.24 ± 0.00 ^d^	3.8	1.11 ± 0.00 ^a^	17.6	0.90 ± 0.00 ^b^	8.6
Total phenolic by MS *	24.39 ± 0.17 ^c^	49.5	45.19 ± 0.37 ^a^	80.2	17.22 ± 0.73 ^d^	39.8	34.32 ± 0.25 ^b^	52.1	42.63 ± 0.53 ^a^	65.3
Total phenolic by FC **	65.19 ± 3.26 ^d^	99.6	187.61 ± 4.81 ^a^	136.1	43.34 ± 2.26 ^e^	117.8	102.92 ± 1.74 ^c^	125.7	127.66 ± 3.92 ^b^	114.2

Different letters within the same row mean significant different (*p* < 0.05) values. n.d. means that the compound was not detected in the sample. * Total phenolic compounds by mass spectrometry. Sum of the amount of individual phenolic compounds. ** Total phenolic compounds by Folin-Ciocalteu’s assay. Data are expressed as mg of gallic acid equivalent per 100 g of raw or cooked onion.

## Data Availability

The data presented in this study are available in here and in supplementary material.

## Supplementary Materials

The following are available online at https://www.mdpi.com/article/10.3390/foods10051023/s1, Table S1: Mass spectral data of phenolic compounds identified in onion samples in negative ionization mode, Table S2: Mass spectral data of phenolic compounds identified in onion sample in positive ionization mode.
