# Supplementary material for: Influence of Cooking Methods on Onion Phenolic Compounds Bioaccessibility
Source: Foods. 2021 May 8;10(5):1023. doi: 10.3390/foods10051023 (PMC8151956; doi:10.3390/foods10051023)
Supplement: Supplementary file 1 [file foods-10-01023-s001.zip › Table S1.pdf]

Table S1. Mass spectral data of phenolic compounds identified in onion samples in negative ionization mode.

Rt	Compound	[M-H]$^-$ *(m/z)*	MS^2 ion fragments *(m/z)*
7.7	Protocatechuic acid-hexoside	315	153 (100%), 109 (4%)
9.5	Quercetin-tri-*O*-hexoside isomer	786	625 (100%), 463 (10%), 301 (2%)
9.9	Kaempferol-7-*O*-hexoside isomer	447	285 (100%), 284 (18%), 257 (4%)
9.9	Taxifolin-*O*-hexoside isomer	465	285 (100%), 241 (8%), 303 (5%)
9.9	Kaempferol-hexoside-rhamnoside-rhamnoside	739	447 (100%), 284 (4%), 285 (3%)
10.0	Kaempferol-3-*O*-hexoside-7-*O*-hexoside isomer	609	447 (100%), 285 (4%)
10.1	(Epi)catechin-3-*O*-hexoside isomer	451	289 (100%), 245 (10%), 205 (5%)
10.2	Isorhamnetin-*O*-hexoside-*O*-pentoside	609	315 (100%), 447 (11%)
10.3	Caffeic acid-*O*-hexoside	341	179 (100%)
10.5	Taxifolin-*O*-hexoside isomer	465	303 (100%), 285 (43%), 241 (3%)
10.6	(Epi)catechin-3-*O*-hexoside isomer	451	289 (100%), 245 (16%), 205 (6%)
10.6	Quercetin-3-*O*-hexoside-7-*O*-hexoside	625	463 (100%), 301 (14%)
10.7	Myricetin-*O*-hexoside-*O*-hexoside isomer	641	479 (100%), 317 (11%)
10.8	Taxifolin-*O*-hexoside isomer	465	303 (100%), 285 (45%), 241 (5%)
10.8	Sinapic acid-*O*-hexoside isomer	385	223 (100%), 208 (5%)
11.4	Taxifolin-*O*-hexoside isomer	465	303 (100%), 285 (4%), 241 (4%)
11.5	Ferulic acid-*O*-hexoside	355	193 (100%), 178 (65%)
11.5	Sinapic acid-*O*-hexoside isomer	385	223 (100%), 208 (29%)
11.6	Myricetin-*O*-hexoside-*O*-hexoside isomer	641	479 (100%), 317 (12%)
11.8	Quercetin-7-*O*-hexoside-4’-*O*-hexoside	625	463 (100%), 301 (5%)
12.2	Quercetin-tri-*O*-hexoside isomer	786	625 (100%), 301 (7%), 463 (2%)
12.3	Quercetin-3-*O*-hexoside-4’-*O*-hexoside	625	463 (100%), 301 (9%). 179 (2%)
12.4	Kaempferol-3-*O*-hexoside-7-*O*-hexoside isomer	609	447 (100%), 285 (66%)
12.5	Isorhamnetin-3-*O*-hexoside isomer	477	315 (100%)
12.6	Isorhamnetin-3-*O*-hexoside-4’-*O*-hexoside	639	477 (100%), 315 (4%)
12.8	Taxifolin-*O*-hexoside isomer	465	303 (100%), 285 (93%), 241 (2%)
14.0	Quercetin-3-*O*-hexoside isomer	463	301 (100%), 179 (3%)
14.8	Isorhamnetin-3-*O*-hexoside isomer	477	315 (100%)
15.1	Quercetin-4’-*O*-hexoside	463	301 (100%), 179 (4%), 151 (3%)
15.4	Kaempferol-7-*O*-hexoside isomer	447	285 (100%), 284 (6%), 257 (4%), 151 (3%)
15.5	Isorhamnetin-4’-*O*-hexoside isomer	477	315 (100%)
16.1	Quercetin-3-*O*-glucoside	463	301 (100%), 151 (13%), 179 (4%)

16.1	Kaempferol-3-*O*-hexoside isomer	447	284 (100%), 285 (80%), 255 (5%)
18.1	Quercetin	301	179 (100%), 151 (65%)
