# Supplementary material for: Influence of Cooking Methods on Onion Phenolic Compounds Bioaccessibility
Source: Foods. 2021 May 8;10(5):1023. doi: 10.3390/foods10051023 (PMC8151956; doi:10.3390/foods10051023)
Supplement: Supplementary file 1 [file foods-10-01023-s001.zip › Table S2.pdf]

Table S2. Mass spectral data of phenolic compounds identified in onion sample in positive ionization mode.

Rt	Compound	$[M]^+$ *(m/z)*	MS^2 ion fragments *(m/z)*
9.0	Cyanidin-*O*-hexoside-*O*-hexoside isomer	611	287 (100%), 449 (90%)
9.9	Cyanidin-3-*O*-hexoside	449	287 (100%)
10.0	Cyanidin-*O*-hexoside-*O*-hexoside isomer	611	287 (100%), 449 (20%)
10.5	Peonidin-3-*O*-hexoside	463	301 (100%)
10.7	Cyanidin-*O*-malonyl-hexoside isomer	535	287 (100%)
11.0	Cyanidin-*O*-malonyl-hexoside isomer	535	287 (100%), 449 (5%)
11.1	Cyanidin-*O*-hexoside-*O*-malonyl-hexoside	697	287 (100%)
11.4	Malvidin-*O*-hexoside-acetaldehyde	517	355 (100%), 339 (32%)
11.7	Peonidin-*O*-malonyl-hexoside isomer	549	301 (100%), 463 (3%)
